# A new surgical technique for concealed penis using an advanced musculocutaneous scrotal flap

**DOI:** 10.1186/s12894-015-0044-3

**Published:** 2015-06-19

**Authors:** Dong-Seok Han, Hoon Jang, Chang-Shik Youn, Seung-Mo Yuk

**Affiliations:** The Catholic University of Korea, Daejeon Saint Mary’s Hospital, 64, Daeheung-ro, Jung-gu, Daejeon 301-723 Republic of Korea

**Keywords:** Penis, Scrotum, Lymphedema

## Abstract

**Background:**

Until recently, no single, universally accepted surgical method has existed for all types of concealed penis repairs. We describe a new surgical technique for repairing concealed penis by using an advanced musculocutaneous scrotal flap.

**Methods:**

From January 2010 to June 2014, we evaluated 12 patients (12–40 years old) with concealed penises who were surgically treated with an advanced musculocutaneous scrotal flap technique after degloving through a ventral approach. All the patients were scheduled for regular follow-up at 6, 12, and 24 weeks postoperatively. The satisfaction grade for penile size, morphology, and voiding status were evaluated using a questionnaire preoperatively and at all of the follow-ups. Information regarding complications was obtained during the postoperative hospital stay and at all follow-ups.

**Results:**

The patients’ satisfaction grades, which included the penile size, morphology, and voiding status, improved postoperatively compared to those preoperatively. All patients had penile lymphedema postoperatively; however, this disappeared within 6 weeks. There were no complications such as skin necrosis and contracture, voiding difficulty, or erectile dysfunction.

**Conclusions:**

Our advanced musculocutaneous scrotal flap technique for concealed penis repair is technically easy and safe. In addition, it provides a good cosmetic appearance, functional outcomes and excellent postoperative satisfaction grades. Lastly, it seems applicable in any type of concealed penis, including cases in which the ventral skin defect is difficult to cover.

## Background

Concealed penis (CP) is a congenital abnormality in which the penis is concealed within the subcutaneous tissue [1]. Specifically, the penis appears to be fused to the scrotum, and the penile shaft is entrapped within the subcutaneous tissue.

CP can cause phimosis, balanitis, difficulties with hygiene and voiding, and embarrassment among peers. Since most cases of CP do not spontaneously resolve, surgical correction is recommended, except in cases where CP is secondary to excessive suprapubic or prepubic fat.

In the surgical correction of CP, covering the penile skin defect is a major challenge, but is also the most important part of the procedure. By performing the surgical procedure through a ventral approach, the ventral skin defect is created; therefore, any skin available for covering the ventral defect is deficient in most cases. Covering the ventral skin defect is dependent on the appearance of the penis and whether there is enough elongation without tension during penile erection. Sufficient coverage of the ventral skin defect results in successful, functional, and cosmetic outcomes.

Numerous surgical techniques for correcting CP have been described. However, until recently, no single, universally accepted surgical method has existed for all CP repairs. Occasionally, the skin defect cannot be covered following previous surgical intervention. Therefore, new surgical techniques are warranted to achieve better cosmetic and functional outcomes.

We discuss using the advanced musculocutaneous scrotal flap for covering the ventral skin defect in the correction of CP, and we evaluated the patients’ surgical outcomes.

## Methods

### Subjects

From January 2010 to June 2014, we evaluated 12 cases of CPs that were surgically treated using the advanced musculocutaneous scrotal flap technique after degloving through a ventral approach.

During the preoperative examinations, all patients had the initial appearance of a short penis with a minimal penile shaft skin, and the normal penile shaft was palpated and visualized while applying pressure on both sides of the shaft base.

All patients enrolled in this study did not have appropriate penile skin coverage, according to the methodologies of Sugita et al. [2] and Kim et al. [3] Patients were scheduled for regular follow-up visits at 6, 12, and 24 weeks postoperatively. Data on patients’ age, operative times, postoperative complications, and satisfaction were collected, and retrospective analysis was performed.

### Evaluation of patients’ satisfaction and postoperative complications

We administered a questionnaire on penile size, morphology, and voiding status to evaluate the patients’ satisfaction. The degree of satisfaction was determined on a scale of 1–5 using the following description: grade 1, very unsatisfactory; grade 2, unsatisfactory; grade 3, neither satisfactory nor unsatisfactory; grade 4, satisfactory; and grade 5, very satisfactory. The questionnaire was administered at all follow-ups. Information regarding the complications was obtained during the patients’ postoperative hospital stay and at all follow-ups.

### Surgical technique

**Fig. 1:**
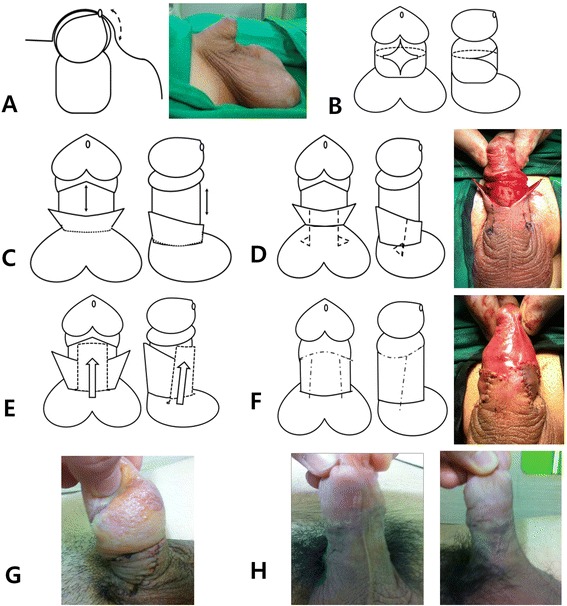
The advanced musculocutaneous scrotal flap technique procedures for correcting concealed penis: **a**) the ventral vertical incision (dotted line); **b**) the fully exposed glans, creation of the diamond-shaped skin defect, and an additional circumferential incision (dotted line); **c**) penile elongation with complete degloving; **d**) the paramedial vertical incision (dotted line) with triangles; **e**) scrotal flap advancement; **f**) the interrupted suture; **g**) postoperative lymphedema; and **h**) the final features of the penis. Written informed consent was also obtained from the patient (s) for publication of any accompanying images

The patient was anesthetized using general endotracheal anesthesia and was positioned supine on the operating table. An incision was made along the ventral midline just proximal to the corona down to the penoscrotal junction in order to create a slit in the phimotic ring of the prepuce (Fig. 1a), and the glans were completely exposed. A diamond-shaped ventral skin defect was created (Fig. 1b), and a circumferential skin incision was made between each side of the diamond-shaped skin defect. Then the penile shaft was completely degloved. Dissection was performed proximally along the Buck’s fascia frees the penis from its deep tetherings to the penile base. Subsequently, the penile shaft became elongated, and the ventral skin defect became increased (Fig. 1c). If it was not possible to cover the ventral skin defect using the redundant dorsal skin according to the methodologies by Sugita et al. [2] and Kim et al., [3] we proceeded with the advanced musculocutaneous scrotal flap technique. We marked and incised two paramedian lines, which extended from the defect to the scrotum and two triangles with bases slightly shorter than the length of the defect (Fig. 1d). After excising both triangles, we mobilized the flap and advanced it over the defect. We confirmed that the skin defect was completely covered, and there was no tension in the suture lines (Fig. 1e).

The dermal tissue at the penopubic junction was sutured to Buck’s fascia at the 11–1 o’clock position, and another dermal tissue flap at the penoscrotal junction was sutured to the fascia at the 5–7 o’clock position. Lastly, interrupted sutures with 5-zero polyglycolic acid were placed (Fig. 1f). No urethral catheter was left, and antibiotic ointment was applied with a dressing.

### Statistical analysis

All statistical analyses were performed using SPSS, version 20.0 for Windows (SPSS Inc., Chicago, IL, USA). The Friedman test was used to compare the changes between the pre- and postoperative satisfaction grades to the time after operation. A p-value <0.05 was considered statistically significant.

### Ethical statement

The study was approved by the Catholic Medical Center Office of Human Research Protection Program of the Catholic University of Korea (DC13OISI0083), and written informed consent was obtained from the patients for participation in this study. For participants aged 16 years, or younger, written consent was obtained for their parents or legal guardian. Written informed consent was also obtained from the patient (s) for publication of any accompanying images.

## Results

The mean age of the patients who underwent operation was 19.9 years (range, 12–40 years), and the mean follow-up period was 27.4 months (range, 10–46 months). The patients’ satisfaction grades, including the penile size, morphology, and voiding status, were improved postoperatively compared to those preoperatively. These improvements became clearer after 6 weeks and lasted after 24 weeks of follow-up (Table 1).

**Table 1:** Patients’ demographics, satisfaction grade, and surgical complications

Mean age (years)			19.86 ± 12.63 (range, 12–41)			
Operative time (mins)			130.08 ± 3.54			
Mean follow up (months)			27.44 ± 12.63			
Patient satisfaction grade						
	Pre-OP	Post-Op	6 weeks	12 weeks	24 weeks	p-value
Penile size	1.42 ± 0.51	3.66 ± 0.49	4.75 ± 0.45	4.67 ± 0.49	4.75 ± 0.45	0.000
Penile morphology	1.33 ± 0.49	3.91 ± 0.29	4.67 ± 0.49	4.42 ± 0.51	4.41 ± 0.51	0.000
Voiding status	1.83 ± 0.72	4.00 ± 0.43	4.41 ± 0.52	4.45 ± 0.52	4.41 ± 0.51	0.000
Complications (n)						
	Post-Op		6 weeks	12 weeks	24 weeks	
Lymphedema	12		0	0	0	
Skin necrosis	0		0	0	0	
Tissue contracture	0		0	0	0	
Wound infection	1		0	0	0	

Pre-op, preoperatively; Post-op, postoperatively

All patients had penile lymphedema postoperatively (Fig. 1g); however, it disappeared within 6 weeks (Fig. 1h). Additionally, postoperative wound infection occurred in one patient, and it was resolved by performing daily sterile dressings and administering oral antibiotics for 2 weeks.

There were no complications such as skin necrosis, tissue contracture, voiding difficulty, or erectile dysfunction. In one patient, temporary discoloration developed on the distal end of the dorsal skin, which was not a part of the advanced musculocutaneous scrotal flap; however, this resolved spontaneously after 2 weeks.

## Discussion

CP results from a combination of deficient penile skin, poor attachment of the penile skin to the deeper layers at the base of the penis, dartos tethering, and sometimes excessive suprapubic fat [4]. It appears that a skin defect of the penile shaft is due to abnormal attachment of the dartos muscle to the corporal bodies during penile development. These abnormal fibromuscular attachments result in tethering of the penile shaft skin to the abdominal wall, which prevents normal skin development [4].

To correct CP, numerous surgical techniques have been described with various surgical outcomes. During the surgical correction of CP, there are four important steps to follow: [5] 1) the penis must be mobilized/elongated by completely degloving it at its base; 2) the dermis and dartos fascia must be secured to the deeper fascia; 3) the penopubic and penoscrotal angles must be restored; and 4) the preputial skin must be re-established to provide skin cover. A deficiency in the penile shaft skin usually occurs because of lack of skin development.

In various surgical techniques for correcting CP, Wollin et al. [6] introduced a surgical method via a ventral approach in 1990. Among the surgical techniques using the ventral approach, the methodologies by Sugita et al. [2] and Kim et al. [3] are useful in correcting CP. The major difference between these method is which skin flap is used for the penile skin defect. In Sugita et al.’s method, the Byars flap is transpositioned using an inverted T-shaped incision on the distal dorsal prepuce, which is used for covering the ventral skin defect. In Kim et al.’s method, the Byars flap is transpositioned using a longitudinal incision on the proximal dorsal prepuce, which is used for covering the ventral skin defect.

However, there is an important pre-condition in their surgical procedures. For functional and cosmetic success, it is imperative to have a sufficient length of the dorsal prepuce to cover the ventral skin defect, because the shaft skin defect occurs after penile degloving and elongation. If there is excessive penile elongation, the ventral skin defect will not be covered by Sugita et al. and Kim et al.’s methods. Therefore, in a case where the dorsal prepuce is insufficient or limited for penile lengthening, it is necessary to use a new method to cover the ventral skin defect.

We used an advanced musculocutaneous scrotal flap for covering the ventral skin defect after penile degloving through a ventral approach during the surgical correction of CP. The scrotum has three blood supplies (i.e., anterolaterally, the superficial and deep external pudendal arteries; posteriorly, the perineal branches of the internal pudendal arteries and deep layer; and the cremasteric branch of the inferior epigastric arteries and branches of the testicular arteries) [7]. Additionally, the scrotum has more tissue elasticity. Therefore, scrotal flaps have been used to cover the ventral surface of the penis after urethroplasty [8–10]. In addition, scrotal advancement [10] and transposition [11] flaps have been used to cover the base of the denuded penis. Advantages of scrotal flaps include the ease and rapidity with which they can be elevated, the fact that they are directly adjacent to the penis, and that the scrotal donor site can be closed primarily without difficulty [12].

In our surgical method, the dorsal penile skin defect that occurred during penile elongation was covered by the dorsal prepuce. Therefore, the ventral skin defect was covered by our advanced musculocutaneous scrotal flap. It was sufficient to cover the defect using the dorsal penile skin, because the dorsal prepuce was not used for the Byars flap transposition. There was no size limitation in covering the ventral skin defect by using our advanced musculocutaneous scrotal flap because of its better tissue elasticity and abundant blood supply. Furthermore, the definite penoscrotal junction can be made using an artificial triangle excision, which has a better cosmetic result.

Although CP can cause hygiene problems, voiding difficulties, and embarrassment among peers, it is not a life-threatening disease. Therefore, the success or failure of the operation was decided based on the satisfaction of the patients and their families, and their satisfaction was based on the functional and cosmetic outcomes. Previous studies have shown that patients and families are generally satisfied with the surgical results [2, 13] and would recommend the surgery to a friend whose child had a similar condition [3].

Our surgical method showed a satisfaction rate that was comparable to findings from previous studies on penile size, morphology, and voiding status. However, there was a difference between previous reports and our study. In our study, the median age at surgery was higher (19.9 years) than that of previous reports, including Sugita et al. (33 months) [2] and Kim et al. (4.7 years) [3]. During the interviews with patients and their families, we found that the patient’s parents lacked the medical knowledge on CP. Therefore, they visited the hospital only when the patient recognized his penis abnormality compared to others, and appealed to their parents about this difference. Additionally, adult patients found it difficult to visit the hospital because of their sexual humiliation associated with hygiene problems, abnormal penile appearance, and embarrassment. Although the median age at surgery was higher than that of previous reports, we evaluated the postoperative satisfaction directly from most of the patients, not from their parents and families.

The mean follow-up period was 27.44 months, and patient satisfaction improved postoperatively compared to that preoperatively in our study. This improvement became more definite after 6 weeks and lasted after 24 weeks of follow-up. We thought that a more improved satisfaction after 6 weeks compared to the postoperative state would be a result of a definite penile appearance after resolving penile lymphedema.

Postoperative complications associated with the correction of CP such as lymphedema [14–16], skin necrosis, and tissue contracture were reported [2]. After performing our surgical method, postoperative lymphedema occurred in all patients and it seemed to be caused by circumferential dissection during complete degloving along Buck’s fascia. However, it resolved spontaneously by 6 weeks postoperatively. Additionally, there was no skin necrosis and tissue contracture. We thought that this result was based on the abundant blood supply and elasticity of the advanced musculocutaneous scrotal flap.

There are some disadvantages to our surgical method. First, most patients mentioned the differences in the skin properties and the scrotal hair bearing on the penile shaft. However, these changes to the penile shaft skin were limited to the part of the entire penile shaft, and most of the patients considered this condition to be a tolerable inconvenience, compared to satisfactory surgical results, such as a normalized penile appearance, increased penis size, and restoration of sexual self-confidence. Second, a bilateral incision scar was made in our surgical method. However, it was not considered to be serious according to the patients’ satisfaction.

## Conclusions

Our advanced musculocutaneous scrotal flap technique for correcting CP is technically easy and safe. Our surgical method provided patients with a good cosmetic appearance, functional outcomes, and excellent postoperative satisfaction grades. Additionally, it seems applicable in any type of CP, including cases in which covering the ventral skin defect is difficult.
